# Nasal Swab Performance by Collection Timing, Procedure, and Method of Transport for Patients with SARS-CoV-2

**DOI:** 10.1128/JCM.00569-21

**Published:** 2021-08-18

**Authors:** Cody Callahan, Rose A. Lee, Ghee Rye Lee, Kate Zulauf, James E. Kirby, Ramy Arnaout

**Affiliations:** a Department of Radiology, Beth Israel Deaconess Medical Center, Boston, Massachusetts, USA; b Department of Pathology, Beth Israel Deaconess Medical Center, Boston, Massachusetts, USA; c Division of Infectious Diseases, Department of Medicine, Beth Israel Deaconess Medical Center, Boston, Massachusetts, USA; d Harvard Medical School, Boston, Massachusetts, USA; e Department of Surgery, Beth Israel Deaconess Medical Center, Boston, Massachusetts, USA; f Division of Clinical Informatics, Department of Medicine, Beth Israel Deaconess Medical Center, Boston, Massachusetts, USA; UNC School of Medicine

**Keywords:** SARS-CoV-2, COVID-19, nasal swab, NP swab, limit of detection

## Abstract

The urgent need for large-scale diagnostic testing for SARS-CoV-2 has prompted interest in sample collection methods of sufficient sensitivity to replace nasopharynx (NP) sampling. Nasal swab samples are an attractive alternative; however, previous studies have disagreed over how nasal sampling performs relative to NP sampling. Here, we compared nasal versus NP specimens collected by health care workers in a cohort of individuals clinically suspected of COVID-19 as well as SARS-CoV-2 reverse transcription (RT)-PCR-positive outpatients undergoing follow-up. We compared subjects being seen for initial evaluation versus follow-up, two different nasal swab collection protocols, and three different transport conditions, including traditional viral transport media (VTM) and dry swabs, on 307 total study participants. We compared categorical results and viral loads to those from standard NP swabs collected at the same time from the same patients. All testing was performed by RT-PCR on the Abbott SARS-CoV-2 RealTime emergency use authorization (EUA) (limit of detection [LoD], 100 copies viral genomic RNA/ml transport medium). We found low concordance overall, with Cohen’s kappa (κ) of 0.49, with high concordance only for subjects with very high viral loads. We found medium concordance for testing at initial presentation (κ = 0.68) and very low concordance for follow-up testing (κ = 0.27). Finally, we show that previous reports of high concordance may have resulted from measurement using assays with sensitivity of ≥1,000 copies/ml. These findings suggest nasal-swab testing be used for situations in which viral load is expected to be high, as we demonstrate that nasal swab testing is likely to miss patients with low viral loads.

## INTRODUCTION

Controlling the COVID-19 pandemic will require a massive expansion of testing for SARS-CoV-2. Until recently, nasopharyngeal (NP) swabs were the U.S. Centers for Disease Control and Prevention’s (CDC) preferred specimen type, as these specimens were thought to provide the most robust detection of patient infection. However, there are conflicting reports as to which of several specimen types bear the highest viral load (1–3), and ultimately, the “preferred-specimen” specification was removed from interim CDC guidance on 29 April 2020 (4). Sensitivity is a complex issue, however, as detection in the upper airways (nasopharynx and oropharynx) is affected by multiple factors, including duration of illness prior to testing (5) and the limit of detection (LoD) of the reverse transcription (RT)-PCR assay used (6).

Availability of NP swabs and the resources to establish NP collection sites with specimen collection personnel have remained critical bottlenecks. To resolve these issues, health care systems have adopted multiple different strategies, including engaging industrial manufacturers to mass produce novel 3D-printed NP swabs (7), as well as evaluating different specimen types and alternative sample collection strategies (8–16). Assessment of nasal swabs is a rapidly growing area of interest, specifically because this specimen type involves a less invasive procedure than NP swabs. Accordingly, such samples can be self-collected by patients with a simple set of instructions, alleviating the need for medical personnel for specimen collection and reducing use of personal protective equipment (PPE) in short supply.

Many of the U.S. Food and Drug Administration emergency use authorization (FDA EUA) RT-PCR assays have approval for use of nasal swabs as a specimen type, but how well nasal swabs perform compared to NP swabs remains unclear. Recommendations by the Infectious Disease Society of North America caution that levels of evidence are low. To date, nasal swab studies have shown conflicting results, with some researchers reporting similar test performance to NP swabs and others finding decreased sensitivity (8, 10, 12–16). Reconciling these differences is challenging, as these studies employed different sampling materials, collection methods, and RT-PCR assays. To address these conflicting reports, here we describe the results of a multiarm, 308-subject study comparing sampling in two different clinical scenarios (initial presentation versus follow-up), two different health care worker nasal swab collection procedures, and three different transport conditions, including in viral transport media (VTM) and dry transport. We discuss our findings in the context of prior reports to more systematically assess nasal swab test performance and its preferred role(s) in addressing diagnostic and epidemiologic needs in the COVID-19 pandemic.

## MATERIALS AND METHODS

### Trial design.

This was a multiarm study involving initial versus follow-up presentation, three different specimen-transport conditions, and two collection procedures, using a standard NP swab as a control.

### Transport conditions and swabs used.

Standard nasal swabs (see immediately below) were compared for subjects presenting for their first COVID-19 test versus subjects with a previous test presenting for follow-up, collected via a shallower/shorter versus a deeper/longer collection method (see Fig. S1 in the supplemental material), under three different specimen-transport conditions: (*i*) a guanidine thiocyanate (GITC) transport buffer, part of the Abbott Multi-Collect specimen collection kit (catalog no. 09K12-004; Abbott Laboratories, Abbott Park, IL), (ii) dry, with no buffer, and (iii) in modified CDC viral transport medium (VTM) (Hanks’ balanced salt solution containing 2% heat-inactivated fetal bovine serum [FBS], 100 μg/ml gentamicin, 0.5 μg/ml fungizone, and 10 mg/liter Phenol red, produced by the Beth Israel Deaconess Medical Center [BIDMC] Clinical Microbiology Laboratories [17]). The nasal swab used was the included Abbot swab for the GITC arm and the Hologic Aptima multitest swab otherwise (catalog no. AW-14334-001-003; Hologic, Inc., Marlborough, MA), all with polyester/nylon/rayon spun material. The NP swab used was the Copan BD ESwab collection and transport system swab, with a head of flocked nylon (catalog no. 220532; Copan Diagnostics, Inc., Murietta, CA). (Note that only the NP swab from the Copan kit was used: the transport medium for the NP swab was 3 ml VTM, not the 1 ml liquid amies transport medium that is part of that kit; see “Swab Collection Protocols,” below.).

### Participants and collection.

Participants were adults over 18 years of age tested for SARS-CoV-2 during the normal course of clinical care, based either on clinically suspected COVID-19 infection or follow-up after previous SARS-CoV-2-positive RT-PCR testing. Participants were asked to be swabbed twice, first with one of the nasal swabs under study (see below for swab collection protocols) and then with a standard NP swab. To control for potential variability related to self-swabbing, sample collection was performed by trained nurses or respiratory therapy staff (“study staff”) with training and oversight from the respiratory therapy department at Beth Israel Deaconess Medical Center (BIDMC) drive-through/walk-up (“drive-through”) COVID-19 testing sites. Individuals with known thrombocytopenia (<50,000 platelets/μl) were excluded from the study to avoid risk of bleeding. This study was reviewed and approved by BIDMC’s institutional review board (IRB protocol no. 2020P000451).

### PCR compatibility.

Although all of the above swabs are routinely used for PCR testing, as a double-check, each swab type was assessed for PCR compatibility by overnight incubation in 3 ml of modified CDC VTM (allowing potential PCR inhibitors time to leech into media), spiking 1.5 ml of medium with 200 copies/ml of control SARS-CoV-2 amplicon target (twice the LoD of our system), vortexing, and testing using the Abbott RealTime SARS-CoV-2 assay on an Abbott m2000 RealTime system platform (18), the assay and platform used for all testing in this report, following the same protocol used for clinical testing (see below). All swabs examined in this study passed this quality-control testing for lack of RT-PCR inhibition based on observation of cycle threshold (*C_T_*) values within expected quality control limits (17).

### Swab collection protocols.

For the shallower/shorter collection procedure (henceforth, “shallow”), for each naris, the swab tip was inserted into the nostril, the patient was told to press a finger against the exterior of that naris, and the swab was rotated against this external pressure for 10 seconds; this procedure was repeated with the same swab on the other naris, and then the swab was placed into the collection tube for transport to the laboratory for testing (Fig. S1a). For the deeper/longer collection procedure (henceforth, “deep”), the swab was inserted into the naris until resistance was felt, and the swab was then rotated for 15 seconds without external pressure (Fig. S1b); this procedure was repeated with the same swab on the other naris, and the swab was then placed into the collection tube for transport (15). The NP swab sample was collected from a single naris by standard technique: insertion to appropriate depth, 10 rotations regardless of time, removal, and placement into a transport medium tube containing 3 ml of VTM (4). To maximize collection of material from the nares, in all cases, sampling using the nasal swab (both nares) was performed first, before the NP swab.

### Sample processing and testing.

Samples were sent to the BIDMC Clinical Microbiology Laboratories for testing. Dry swabs were eluted in 2 ml of Abbott mWash1, which consists of 100 mM Tris with guanidinium isothiocyanate (GITC) and detergent. Swabs transported in GITC buffer were supplemented with 1 ml of Abbott mWash1 solution at the lab in order to achieve minimum volume requirements for testing, for a final volume of 2 ml. The NP swab and final nasal swab were each transported (separately) in 3 ml VTM. The length of time between collection and processing was the same, 4 to 14 h for each pair of NP/nasal swabs from the same subject. Tests were performed with 1.5 ml of sample medium (1.5 ml/2 ml = 75% or 1.5 ml/3 ml = 50% of the total medium in the tubes) using the Abbott RealTime SARS-CoV-2 assay for EUA for use with nasopharyngeal and nasal swabs (18). This dual-target assay detects both the SARS-CoV-2 RdRp (RNA-dependent RNA polymerase) and N (nucleocapsid) genes with an in-lab-verified LoD of 100 copies/ml (17, 19).

### Statistical analyses.

For concordance testing, RT-PCR results were considered categorically either positive, if above the reporting threshold of 31.5, or negative; testing was performed using Cohen’s kappa (*κ*) (20).

For analyses based on cycle-threshold (*C_T_*) values, for discordant samples (positive nasal swab/negative NP swab result or vice versa), the negative result was assigned a *C_T_* value of 37, the total number of cycles run. Conversion to viral load was performed as described previously (19).

### Significance testing.

We tested whether *C_T_* values for a given set of nasal swabs differed from the *C_T_* values for the paired NP swabs (the controls) using Wilcoxon’s paired *t* test. This tested the null hypothesis that values for controls and prototypes are drawn from the same underlying distribution. The false-discovery rate (FDR) was used to account for multiple testing, with a significance threshold of α = 0.01.

To test whether the *κ* for a given subgroup of size *n* differed from that of a larger group, we bootstrapped by randomly sampling *n* datapoints from the larger group, calculating *κ* for that randomly sampled subset, and repeating this process 10,000 times to generate a distribution (histogram) of *κ* values; this distribution constitutes a null model of the *κ* one would expect to observe by chance in a sample of *n* results, given the data in the larger group. Using this distribution, we then calculated the probability of observing a *κ* at least as high as the *κ* actually observed for the *n* datapoints in the given subgroup, to test for consistency with expectation. We again used FDR; inconsistency (*P* < 0.05 or *P > *0.95) would reject the null hypothesis that the study arm and the larger pool are statistically indistinguishable (as measured by kappa). For completeness, we performed the same bootstrap analysis to compare procedure 1 (shallow) and procedure 2 (deep) results to all results.

We used Python v3.6-3.8 and its NumPy, SciPy, Matplotlib, Pandas, and ct2vl libraries for the above-described analyses and related visualizations.

### Literature review.

We searched PubMed and the preprint servers bioRxiv and medRxiv through 1 June 2020 for all literature on nasal swab sampling for SARS-CoV-2 and extracted sample sizes, collection methods, RT-PCR assay information, and 2 × 2 contingency table data comparing nasal swabs to NP swabs wherever available.

### Data availability.

All *C_T_* values are available upon request. Conversion from *C_T_* value to viral load for the assay and platform we used is available via the ct2vl Python 3 library, which can be downloaded/installed from PyPI at https://github.com/ArnaoutLab/ct2vl. PHI-scrubbed data and analysis code can be found at https://github.com/rarnaout/Covid_diagnostics/tree/main/Covid_Nasal_SI.

## RESULTS

**FIG 1:**
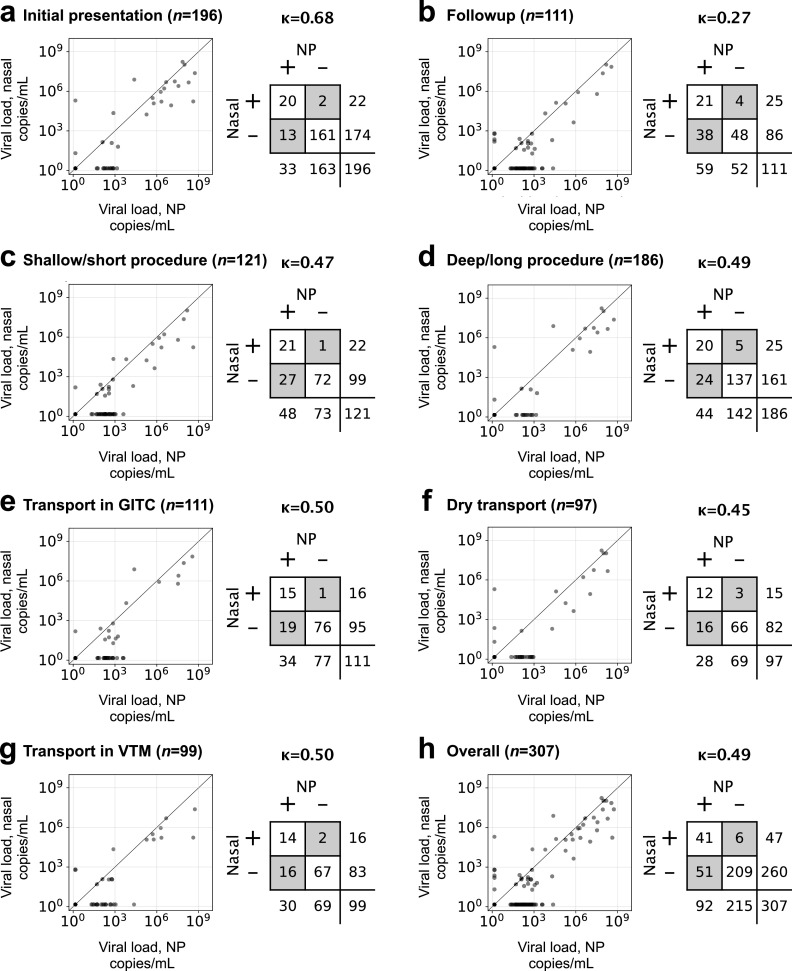
Viral loads for NP (*x* axes) versus nasal swab (*y* axes) for (a) initial versus (b) follow-up testing, (c) shallow/short versus (d) deep/long collection procedures, collection (e) in GITC versus (f) dry versus (g) in VTM, and (h) for all data, with 2 × 2 tables and concordance values measured by Cohen’s kappa, κ. In each plot, the diagonal is a 1:1 line. Dots along the bottom and left axes are negatives.

Table 1 shows the numbers of patients tested in each of the six arms of our nasal versus NP swab study. Visual inspection of plots of the *C_T_* values of the nasal swab versus NP swab controls suggested worse performance for nasal swabs across all six arms, with no obvious differences between the two swab procedures or among the dry swab, VTM, or GITC collection methods (Fig. 1). Statistical testing confirmed that results for each arm were indistinguishable from the overall results, supporting the functional equivalence of all swab/transport-condition combinations (Table 1). The only exception was for comparisons involving initial testing, for which *C_T_* values were lower than for the overall data set and lower than in follow-up testing (5 to 30 days after the initial test; Fig. S2). For concordant positives (*n *= 41), comparison of *C_T_* values between nasal and NP swabs showed higher *C_T_* values for nasal swabs than for NP swabs, suggesting slightly but consistently lower yield from the nasal swabs (Wilcoxon *P < *0.0001). Consistent with this conclusion, there was a marked increase in false negatives for NP swabs with higher *C_T_* values (lower viral loads), resulting in low concordance overall (Cohen’s kappa = 0.49) (Fig. 1).

**TABLE 1:** Overall and subset analysis^*a*,^^*b*^

Analysis	Transport	Timing	Procedure	*n*	TP	FN	FP	TN	Mean *C_T_* value, NP	Mean *C_T_* value, nasal	*P* value, difference of *C_T_*s	*κ*	*P* value, difference of *κ*s
Overall	All	All	All	307	41	51	6	209	20.04	17.36	**0.00026**	0.49	
By timing^*c*^	Initial	All	All	196	20	13	2	161	16.22	13.80	0.033	0.68	**<0.0001**
	Follow-up	All	All	111	21	38	4	48	22.18	20.50	0.0019	0.27	0.0025
By procedure^*c*^	All	Shallow	All	121	21	27	1	72	20.88	18.63	0.0063	0.47	0.40
	All	Deep	All	186	20	24	5	137	19.13	16.25	0.014	0.49	0.43
By transport method^*c*^	All	All	GITC	111	15	19	1	76	20.65	19.15	0.06	0.50	0.39
	All	All	Dry	97	12	16	3	66	18.76	14.89	0.064	0.45	0.32
	All	All	VTM	99	14	16	2	67	20.55	17.90	0.013	0.50	0.39
By timing and procedure^*d*^	Initial	Shallow	All	61	6	2	0	53	14.39	13.88	0.31	0.84	**0.0004**/0.070
	Initial	Deep	All	135	14	11	2	108	16.81	13.77	0.091	0.63	0.011/0.15
	Follow-up	Shallow	All	60	15	25	1	19	22.18	20.40	0.012	0.25	0.023/0.40
	Follow-up	Deep	All	51	6	13	3	29	22.18	20.66	0.09	0.25	0.039/0.41
By procedure and transport method^*e*^	All	Shallow	GITC	46	9	11	1	25	21.08	19.87	0.20	0.44	0.38/0.42
	All	Shallow	Dry	36	5	7	0	24	18.87	15.17	0.06	0.49	0.46/0.39
	All	Shallow	VTM	39	7	9	0	23	22.14	19.31	0.22	0.48	0.50/0.44
	All	Deep	GITC	65	6	8	0	51	20.03	17.95	0.44	0.54	0.29/0.30
	All	Deep	Dry	61	7	9	3	42	18.68	14.74	0.69	0.42	0.29/0.29
	All	Deep	VTM	60	7	7	2	44	18.74	16.80	0.031	0.52	0.38/0.40
By timing and transport method^*d*^	Initial	All	GITC	71	3	6	0	62	20.72	14.97	1.00	0.47	0.44/0.034
	Initial	All	Dry	65	8	3	2	52	13.37	13.64	0.15	0.72	0.012/0.40
	Initial	All	VTM	60	9	4	0	47	15.52	13.59	0.13	0.78	0.0038/0.19
	Follow-up	All	GITC	40	12	13	1	14	20.62	20.11	0.027	0.36	0.20/0.18
	Follow-up	All	Dry	32	4	13	1	14	22.24	17.38	0.38	0.16	0.033/0.20
	Follow-up	All	VTM	39	5	12	2	20	24.40	23.43	0.062	0.22	0.049/0.32
By timing, procedure, and transport method^*c*^	Initial	Shallow	GITC	22	0	1	0	21	22.25			0.00	0.0011
	Initial	Shallow	Dry	20	2	0	0	18	11.31	13.59	0.50	1.00	**<0.0001**
	Initial	Shallow	VTM	19	4	1	0	14	14.04	14.03	0.62	0.85	0.0049
	Initial	Deep	GITC	49	3	5	0	41	20.53	14.97	1.00	0.50	0.31
	Initial	Deep	Dry	45	6	3	2	34	13.83	13.65	0.44	0.64	0.080
	Initial	Deep	VTM	41	5	3	0	33	16.44	13.25	0.12	0.73	0.010
	Follow-up	Shallow	GITC	24	9	10	1	4	21.02	19.87	0.20	0.16	0.0048
	Follow-up	Shallow	Dry	16	3	7	0	6	20.38	16.23	0.25	0.24	0.066
	Follow-up	Shallow	VTM	20	3	8	0	9	25.82	26.36	0.25	0.25	0.050
	Follow-up	Deep	GITC	16	3	3	0	10	19.36	20.93	0.25	0.56	0.26
	Follow-up	Deep	Dry	16	1	6	1	8	24.91	19.12	1.00	0.03	0.077
	Follow-up	Deep	VTM	19	2	4	2	11	21.80	21.23	0.50	0.20	0.018

a TP, true positives; FN, false negatives; FP, false positives; TN, true negatives. Mean *C_T_* values are for true (concordant) positives.

b Bold values indicate statistical significance (*P* value of 0.01 corrected for false-discovery rate using the Benjamini-Hochberg method).

c *P* values for difference of κs are comparing the given row to all data.

d *P* values for difference of κs are comparing the given row to all data/data with the same timing (separated by a slash).

e *P* values for difference of κs are comparing the given row to all data/data with the same procedure (again separated by a slash).

Our finding of low overall concordance was in contrast to some previous reports which found nasal swab collection to exhibit excellent sensitivity as well as *C_T_*-value concordance (13, 15), but was consistent with others (14, 21), including, for example, one recent study at a New York, USA, hospital that also noted lower nasal swab concordance for higher *C_T_* values (16). Close review of these previous reports revealed that they differed in the type of specimen and/or result they used as a reference (e.g., any test-sample positive versus using NP swabs as the gold standard) and in the parameters they used in order to describe test performance (e.g., positive percent agreement versus sensitivity). To control for at least the latter, we extracted 2 × 2 contingency-table data from these reports to facilitate comparison to each other and to our own results (Table 2; Table S1). Notably, many of these studies used a modified version of the CDC assay that did not report a LoD. Furthermore, of the studies that report the *C_T_* values of their results, no viral-load conversion was provided, which is important since different RT-PCR assays and platforms have unique conversions between *C_T_* value and viral load. Therefore, we were unable to systematically compare nasal-swab performance at low viral loads in these reports. These differences left open the possibility that inconsistent comparative performance of nasal-swab sampling might be explained largely by differences in assay LoD, and possibly also by patient viral load. Nasal-swab sampling protocols and transport medium conditions varied between studies; there was no obvious correlation between concordance and whether specimens were collected by the subjects themselves or by health care workers, or the relative timing of collection.

**TABLE 2:** Nasal swab studies with ≥30 SARS-CoV-2-positive subjects

Study	Samples^*a*^	Collection procedure	Self-collected or health care worker-collected	Nasal and NP swabs collected simultaneously	No. of samples from:				Kappa	RT-PCR method (LoD in copies/ml)
					Nasal + NP+	Nasal + NP–-	Nasal – NP+	Nasal – NP–		
Tu et al. (15)	498 individuals tested at 5 different ambulatory centers	Nasal swabs collected with a foam swab (Puritan 25-1506 1PF100) via inserting in the vertical position into one nasal passage until gentle resistance and leaving the swab in place for 10–15 s and rotating. Swabs were stored in viral transport medium.	Self-collected	Yes	47	1	3	447	0.96	Quest Diagnostics SARS-Cov-2 RNA, qualitative real-time RT-PCR (San Juan Capistrano, CA) targeting N1 and N3 (nucleocapsid) genes (LoD 136 cp/ml)
		Midturbinate swabs collected with a nylon flocked swab (MDL NasoSwab A362CS02) via inserting in the horizontal position into the nasal passage until gentle resistance is met, leaving the swab in for 10–15 s and rotating.			50	2	0	452	0.98	
Basu et al. (16)	101 samples collected in an adult ED	Dry nasal samples were obtained with swabs supplied with the Abbott assay (Puritan Medical Products 25-1506 IPF100). Nasal samples were obtained from both nares.	Health care worker-collected	Yes	17	1	14	69	0.61	Abbott ID NOW (LoD, 125 genome equivalents/ml) for nasal swabs Cepheid Xpert Xpress SARS-CoV-2 test (LoD, 250 copies/ml) for NP swabs
Griesemer et al. (25)	463 subjects from outpatient cohorts	Nasal swabs were collected in molecular transport medium (Longhorn Vaccines and Diagnostics), although swab material and procedure unspecified.	Not specified	Yes	86	0	360	17	0.89	CDC 2019 nCoV real-time RT-PCR diagnostic panel (LoD, 1,000 copies/ml using the CDC assay)
Pinninti et al. (26)	69 paired samples taken from 40 patients	69 paired nasal and NP swabs were collected, but swab material, transport, and procedure were unspecified.	Health care worker-collected	Yes	44	0	12	13	0.54	CDC 2019 nCoV real-time RT-PCR diagnostic panel (LoD, 1,000 copies/ml using the CDC assay)
Péré et al. (27)	44 patients	Nasal and NP swabs were inserted in the nostril until they hit an obstacle (the inferior concha and the back of the nasopharyngeal cavity, respectively), rotated five times and removed. The test was conducted in only one nostril per patient.	Not specified	Yes	33	0	4	7	0.72	Allplex 2019-nCoV assay (Seegene, Seoul, South Korea) (LoD, 4,167 copies/ml)
Hanson et al. (28)	354 patients	Nasal swabs were inserted 1 inch into nostril or until resistance met, rotated 3 times, leaving in place for several seconds, then repeated in second nostril with the same swab. Swabs were then placed in 3 ml of sterile 1× PBS (ARUP Laboratories).	Self-collected	Yes	69	1	11	273	0.90	Hologic Aptima SARS-CoV-2 transcription-mediated amplification (TMA) assay (Hologic, Inc.) (LoD: 0.026 TCID_50_/ml)^*b*^
McCulloch et al. (29)	185 patients from drive-through clinics	Home swab collection kit containing a flocked midnasal swab (Copan FloqSwab 56380CS01, Copan Diagnostics, Murrieta, CA) and universal transport medium (UTM) (Becton Dickinson, Franklin, NJ) were delivered to home within hours of first test, and written instructions on how to perform a self-collected nasal swab (instructions were not specified).	Self-collected	No (within 2-day delay)	28	3	7	140	0.81	CDC 2019 nCoV real-time RT-PCR diagnostic panel (LoD, 1,000 copies/ml using the CDC assay)
Harrington et al. (30)	524 paired clinical samples from ED and urgent care centers	Foam nasal swabs used, but procedure, transport, and materials unspecified	Not specified	Yes	138	2	47	336	0.78	Abbott ID NOW instrument for nasal swabs (LoD, 125 genome equivalents/ml) compared to Abbott RealTime SARS-CoV-2 assay for NP swabs performed on the Abbott m2000 system (LoD, 100 copies/ml)

a ED, emergency department.

b TCID_50_, 50% tissue culture infective dose.

**FIG 2:**
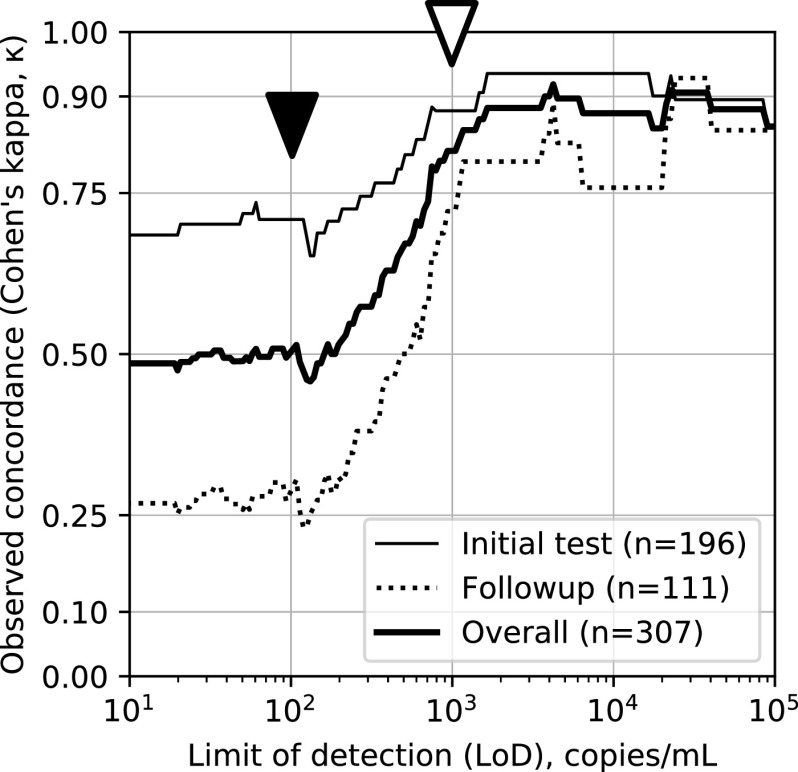
Concordance (measured by Cohen’s kappa [κ]) plotted against assay LoD for all data (thick line), only initial-testing data (thin solid line), and only follow-up-testing data (dotted line). With its LoD of 100 copies/ml (solid arrowhead), the Abbott assay detects false negatives in nasal-swab samples, resulting in low overall concordance (κ = 0.49); even lower concordance for follow-up testing (κ = 0.27), likely because viral loads in this population are lower than they are overall; and still-low concordance for initial testing (κ = 0.71), despite viral loads being higher for initial tests than overall. In contrast, an assay with an LoD of 1,000 copies/ml (open arrowhead) would have missed these false negatives, which would have yielded substantially higher observed concordances regardless of subset.

We therefore revisited the trend we observed of a rise in nasal swab false negatives at higher *C_T_* values (low viral loads). Recently, we demonstrated that *C_T_* values for the SARS-CoV-2 RT-PCR assay and platform used in the present study are reliable quantitative measures of viral load and introduced a conversion from *C_T_* value to viral load (on the Abbott m2000, a viral load of 100 copies/ml corresponds to a *C_T_* value of 26, and 1,000 copies/ml corresponds to a *C_T_* value of ∼21.7) (19). Building on those findings, here, we asked what the concordance would have been, for our nasal versus NP data had the LoD of our assay been higher than its actual 100 copies/ml. Specifically, we recalculated kappa for different LoD cutoffs and found that kappa rose steeply from ∼0.5 (low concordance) to 0.8 to 0.9 (excellent concordance) as the LoD cutoff was increased from 100 copies/ml to 1,000 copies/ml and beyond (Fig. 2). This finding strongly supports the view that nasal swabs miss many if not most patients with low viral load (below ∼1,000 copies/ml) but are reliable for patients with medium or high viral loads, potentially resolving disagreements among previous reports.

## DISCUSSION

Resolving the damage that the COVID-19 pandemic has wrought will require scaling up testing to unprecedented levels. For this reason, there is widespread interest in developing alternatives to NP swab sampling for COVID-19 diagnosis, such as nasal swabs. Proponents argue that the self-administration of these swabs would vastly increase testing capacity, save PPE, and ease the burden on health care workers. Independently, the ability to transport swabs to testing locations without need of transport media such as VTM would further streamline testing processes. Reflecting this interest in nasal swabs, the U.S. CDC has removed the “preference” specification for NP swabs from their interim guidance and note that nasal swabs are an acceptable alternative specimen as of 29 April 2020 (4). However, confidence in population-scale testing strategies based on nasal swabs is complicated by conflicting reports as to how well they perform relative gold standard NP swabs.

We found quite weak concordance between nasal and NP swabs, with Cohen’s kappa values of 0.26 to 0.54 for the six arms and 0.49 overall (Fig. 1), in agreement with some prior studies but in stark contrast with others (Table 2; Table S1). Our results strongly suggest that concordance between nasal and NP swabs depends on the LoD of the PCR assay used to measure positivity, with concordance roughly proportional to viral load; low viral loads may go undetected, depending on the LoD of the assay used. (Fig. 2) (19). We find that nasal swab samples reliably detect patients with viral loads of ≥1,000 copies/ml but miss many patients who have lower viral loads (19). Often, repeat testers present with low viral loads, which may explain the difference in concordance between the initial and follow-up arms of this study and suggests that high-sensitivity assays are necessary to detect viral material in these so-called long-haulers. One possibility is that in cases of high viral load, replicating virus may be more likely to spread to respiratory epithelium bordering and/or in the deeper portions of the anterior nares, where it can be recovered by nasal swab. Note that the expected decrease in reproducibility for viral loads near the limit of detection is insufficient to explain our findings, since if, e.g., nasal and NP sampling were equally sensitive, the decrease in reproducibility would affect them equally, with observations of NP+/nasal− and NP−/nasal+ being equally common, which was not seen.

Our findings may reconcile disagreements in prior reports which have compared nasal swab performance only as a function of *C_T_* values, which are not comparable from study to study, not viral load, as we have done here. We hypothesize that the testing sites in these studies may have selected for patients early in the course of disease, when viral load is high (13, 15). For example, one study (13) that showed high concordance used an assay with a negative *C_T_* cutoff of 40, and only a few patient samples had *C_T_* values above 35. The discrepancy between cutoff and *C_T_* values suggested preferential sampling of patients with only high viral loads. (Note that a *C_T_* value of 35 can correspond to different viral loads in different assays, and the LoD of this assay was 4,167 copies/ml, over 40 times the cutoff in the assay we used.) Notably, the patient population in the present study consisted of both first-time and repeat testing. Many of the latter have been observed to exhibit low-level viral load for weeks in the absence of severe symptoms, and enrichment of these patients may impact the overall performance of NP and nasal swabs in individual studies. In other studies, such differences may be obscured depending on the limit of detection.

Interestingly, we found no difference among transport medium conditions or between sampling protocols, suggesting that the lower sensitivity of nasal swab sampling is an overall limitation of the anatomical location of nasal swab specimens and that the protocols and medium conditions we tested are interchangeable. This was consistent with our review, which demonstrated no obvious correlation between concordance and whether the sample was self-collected or collected by health care workers (which we expect to be roughly bracketed by our two collection procedures). Thus, for patients above a critical threshold of 1,000 copies/ml (Fig. 1), nasal swabs collected in VTM, GITC transport medium, and as dry swabs are all likely to perform equally well in the population, providing multiple potential options for specimen acquisition.

Our results suggest several settings in which nasal swabs may and may not best be used. Peak infectiousness is likely to occur near or shortly before symptom onset (22, 23), and nasopharyngeal viral load is often undetectable a week after symptom onset (2). Lower-sensitivity testing would likely miss patients with early-developing presymptomatic infections and patients presenting multiple days after symptom onset. Notably, for those presenting later to care, a false-negative diagnosis could bear significant clinical implications, erroneously reassuring the patient and clinical team and excluding them from potentially useful and rationed therapies such as remdesivir (24) or others. Importantly, based on viral load distribution in first-time tested individuals at our institution, ∼20% of newly presenting SARS-CoV-2-positive individuals would be missed if sampled solely using nasal swabs (19), highlighting the potential magnitude of this problem.

Nevertheless, nasal swabs provide considerable advantage in terms of ease of collection and potential self-collection. Based on our results, they would serve best in high-test-volume, point prevalence screens in healthy populations, for example, in businesses and universities, where identification of highly infectious individuals will be a prelude to targeted testing with the most sensitive techniques possible to quell outbreaks and forestall local spread. Conversely, nasal swabs should not be used for screening symptomatic and, especially, hospitalized patients, where the more sensitive and resource intensive nasopharyngeal sampling would be justified and help direct care and the most appropriate use of infection control resources. In summary, while nasal swabs are a welcome addition to the armamentarium of tools needed to combat COVID-19, we should be well aware of possible limitations in diagnostic sensitivity and use this resource judiciously.
