# Supplementary material for: Nasal Swab Performance by Collection Timing, Procedure, and Method of Transport for Patients with SARS-CoV-2
Source: J Clin Microbiol. 2021 Aug 18;59(9):e00569-21. doi: 10.1128/JCM.00569-21 (PMC8373031; doi:10.1128/JCM.00569-21)
Supplement: Supplemental file 1 — Fig. S1 and S2, Table S1. Download JCM.00569-21-s0001.pdf, PDF file, 1.3 MB [file jcm.00569-21-s0001.pdf]

Performance of Nasal-Swab Testing for SARS-CoV-2 by Collection Timing, Procedure, and Transport Method

Cody Callahan[a], Rose A. Lee[b,c,d], Ghee Rye Lee[e], Kate Zulauf,[b,d]
James E. Kirby,[b,d] and Ramy Arnaout[b,d,f,#]

Supplementary information

List of figures and files

Supplementary Figure 1. Sample-collection procedures

Supplementary Figure 2. Days after initial presentation among followup specimens

Supplementary Table 1. Comparison of nasal swab studies with less than 30 positive SARS-CoV-2 to date including their collection protocol and RT-PCR assay

Supplementary Figure 1. Sample-collection procedures

a

1. As reasonably needed, tilt the patient's head back to improve access to the anterior nares. For each naris (nostril), perform following.
2. Insert the swab tip into the nostril and press against the inner wall of the naris.
3. Press against the exterior naris, so that you may feel the pressure applied by the swab against your finger.
4. Rotate the swab for **10 seconds**, against the pressure of your finger (clockwise, counterclockwise, or both).
5. Remove swab from first naris and **repeat on the opposite naris**.
6. After thoroughly swabbing each nares, place swab into the appropriate tube.

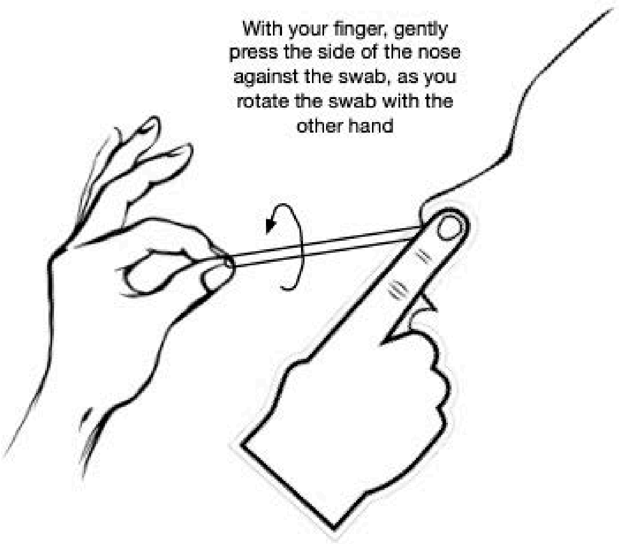

b

1. Insert the swab horizontally into the first nostril until you meet resistance. **The entire length of the bulb should be completely inside the nose.** This might make the participant feel like sneezing; reposition if necessary.
2. **Twirl** the swab around for a full **15 seconds** in the first nostril.
3. Remove the swab from the first nostril and then insert **the same swab** horizontally into the second nostril, again until you meet resistance. Again, **the entire length of the bulb should be completely inside the nose**.
4. **Twirl** the swab around for a full **15 seconds** in the second nostril.
5. Remove swab from nose, place swab into the appropriate tube, and **seal the tube tightly.**

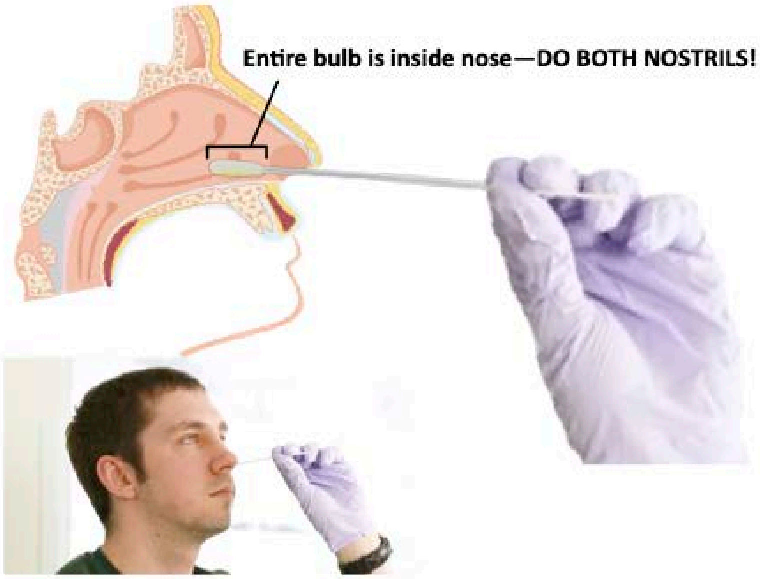

(a) Shallow/short procedure. **(b)** Deep/long procedure.

Supplemental Figure 2. Days after Initial presentation among followup specimens

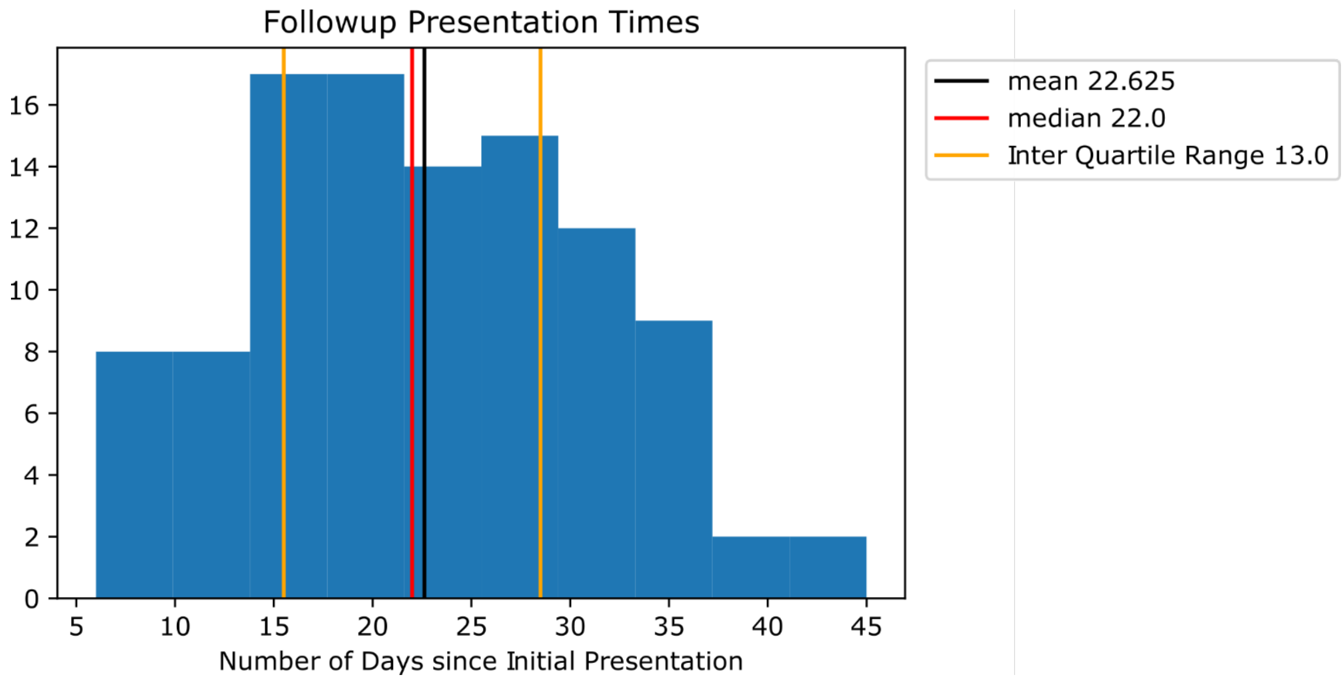

Days after initial presentation among followup (5-30 days since initial presentation) arm.

Supplemental Table 1. Comparison of nasal swab studies with less than 30 positive SARS-CoV-2 to date including their collection protocol and RT-PCR assay

Study	Samples	Collection procedure	Self-collected or Healthcare worker collected	Nasal and NP swabs collected simultaneously	Nasal+/NP+	Nasal+/NP-	Nasal-/NP +	Nasal-/NP-	Kappa	RT-PCR method (LOD in copies/mL)
Berenger et al. (preprint)[1]	36 previously positive patients tested an average of 4d prior	APTIMA Unisex Collection Kit (Hologic Inc.) used to swab both nares to a depth of at least 3 cm (or until resistance felt) and rotated 3 times.	Healthcare worker collected	Yes	22	2	5	7	0.53	Alberta Public Health Laboratory (ProvLab) (LOD: 970 cp/mL)

Minich et al.[2]	10 patients admitted with COVID-19 verified by NP swab	Sterile polyester head, plastic shaft dry swab inserted into one nostril to a depth of ~2-3cm and rotated for 5-10 seconds. Then placed in collection tube containing 0.5 – 1mL 95% ethanol and stored on dry ice.	Not specified	No, but within 72 hrs	3	0*	0**	1	1.00†	CDC protocol adaptation: 4µl RNA template, 100nm forward/reverse primers, 200nm probe, 3µl TaqPath, and water to a 10µl reaction volume performed on the Bio257 rad CFX384 Touch Real-Time PCR Detection System. (no LOD listed)
		Non-sterile cotton head, plastic shaft dry swab, same protocol as above.	Not specified	No, but within 72 hrs	3	0*	1	2	0.66	
		Non-sterile cotton head, plastic shaft dry swab, consumer grade, same protocol as above.	Not specified	No, but within 72 hrs	4	1	1**	1	0.30	
Wehrhahn et al.[3]	236 Australian patients tested at	Nasal swabs were inserted as far as comfortably possible and at least 2–3 cm inside one nostril, rotating the	Both self-collected and healthcare-worker	Yes	17	0	0	219	1.00†	Allplex™ 2019-nCoV Assay (Seegene, Seoul, South Korea) utilising CFX96 Touch RT-PCR Detection

	outpatient locations	swab 5 times and leaving in place for 5–10 seconds.	collected included							Systems (LOD: 4167 copies/mL)
Kojima et al.[4]	43 adults that were recently tested for SARS-CoV-2 via standard NP swab testing	Supervised self-collected nasal swab used a CLASSIQSwab™ that the patient was instructed to insert into one nostril to the depth of 3-4cm and rotate for 5-10 seconds before storing the swab in RNA storage media (DNA/RNA Sheld, Zymo Research Corp)	Self-collected but supervised	Yes	19	4	4***	16	0.63	Modified CDC assay with addition of N3 target to N1, N2 targets. Samples were run on CFX 96™ Touch or Connect Detection System by Bio-Rad (no LOD listed)
Pham et al.[5]	35 paired samples	NS samples were collected first by inserting the swab into the subject's nostril past the inferior turbinate, approximately 3 cm, twisting the swab in the midturbinate area for 3 to 5 s, and placing the swab into a	Not specified	Yes	14	0	0	21	1.0†	SARS CoV-2 TMA assay (LOD: 0.004 TCID50/ml)

		tube of specimen transport media.								

*1 nasal swab was inconclusive, while NP swab was negative, **1 nasal swab was inconclusive, while NP swab positive, ***2 samples were negative due to quantity insufficient. †Undependable given the zeros.

References

1 Berenger B, Fronseca K, Schneider A, Hu J, Zelyas N. Sensitivity of Nasopharyngeal, Nasal and Throat Swab for the Detection of SARS-CoV-2. *Preprint* n.d.

2 Minich JJ, Ali F, Marotz C, Belda-Ferre P, Chiang L, Shaffer JP, *et al.* 2021. Feasibility of using alternative swabs and storage solutions for paired SARS-CoV-2 detection and microbiome analysis in the hospital environment. *Microbiome* **9**:25. https://doi.org/10.1186/s40168-020-00960-4.

3 Wehrhahn M, Robson J, Brown S, Bursle E, Byrne S, New D, *et al.* 2020. Self-collection: An appropriate alternative during the SARS-CoV-2 pandemic. *J Clin Virol* **128**:104417. https://dx.doi.org/10.1016/j.jcv.2020.104417.

4 Kojima N, Turner F, Slepnev V, Bacelar A, Deming L, Kodeboyina S, *et al.* 2020. Self-Collected Oral Fluid and Nasal Swabs Demonstrate Comparable Sensitivity to Clinician Collected Nasopharyngeal Swabs for Coronavirus Disease 2019 Detection. *Clin Infect Dis* 2020:ciaa1589. https://doi.org/10.1093/cid/ciaa1589.

5 Pham J, Meyer S, Nguyen C, Williams A, Hunsicker M, McHardy I, *et al.* 2020. Performance Characteristics of a High-Throughput Automated Transcription-Mediated Amplification Test for SARS-CoV-2 Detection. *J Clin Microbiol* **58**:6. https://dx.doi.org/ 10.1128/JCM.01669-20.
